# Supplementary material for: The interactome of intact mitochondria by cross-linking mass spectrometry provides evidence for coexisting respiratory supercomplexes
Source: Mol Cell Proteomics. 2017 Dec 8;17(2):216–32. doi: 10.1074/mcp.RA117.000470 (PMC5795388; doi:10.1074/mcp.RA117.000470)
Supplement: Supplemental Data [file supp_RA117.000470_133922_0_supp_23978_fzffwf.zip › spectra_annotation/mito_spectra_annotation/107-1-11-1-5-1.pdf]

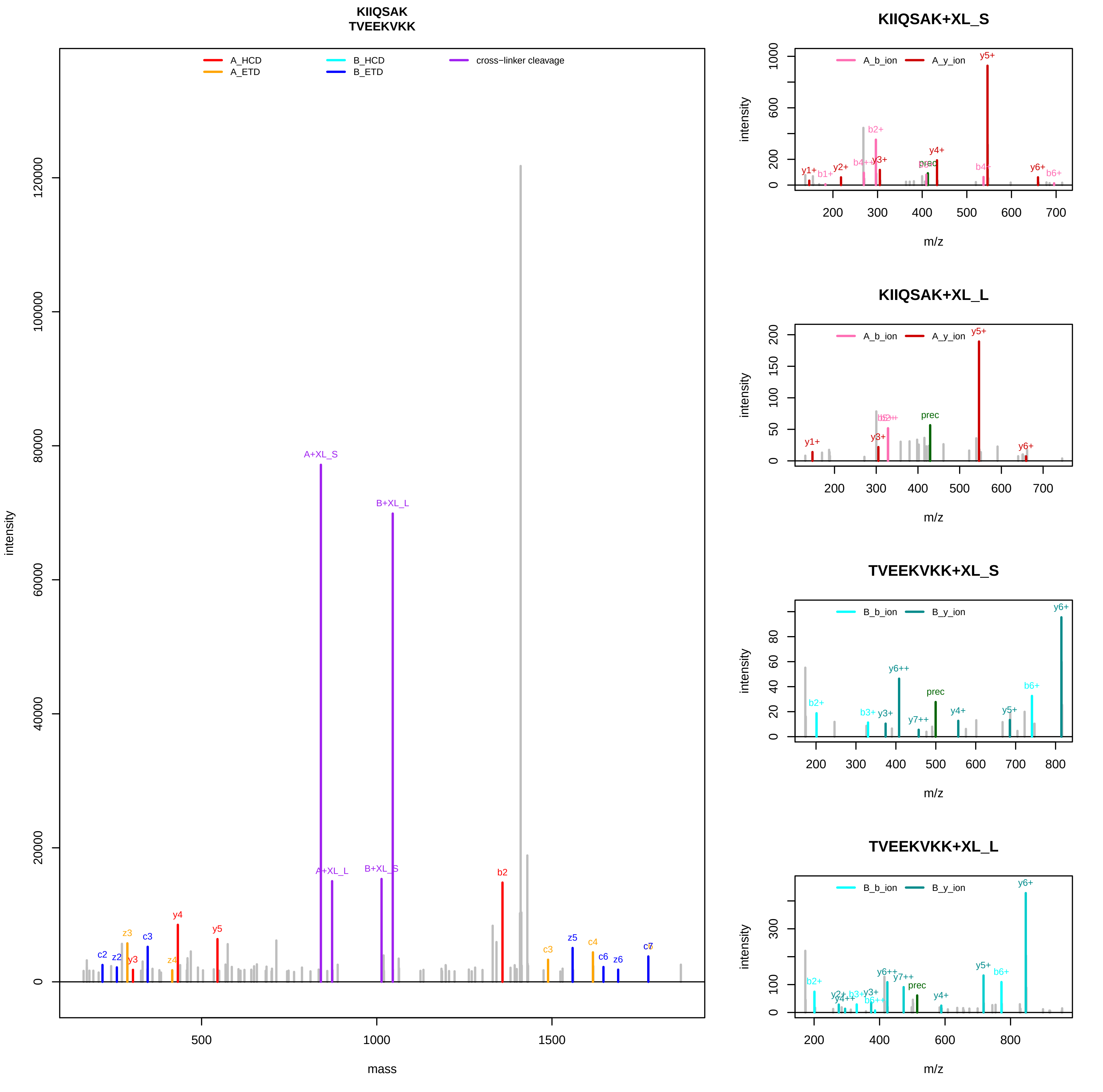

KIIQSAK
TVEEKVKK
A_HCD
A_ETD
B_HCD
B_ETD
cross−linker cleavage
A+XL_S
B+XL_L
A+XL_L
B+XL_S
b2
y4
y5
z3
c3
c2
z2
y3
z4
c3
z5
c4
c6
z6
c7
intensity
mass
KIIQSAK+XL_S
A_b_ion
A_y_ion
y5+
b2+
y4+
y3+
b4++
y1+
b1+
y2+
prec
b4+
y6+
b6+
m/z
KIIQSAK+XL_L
y1+
y3+
b2+
prec
y6+
TVEEKVKK+XL_S
B_b_ion
B_y_ion
y6+
y6++
prec
b6+
b2+
b3+
y3+
y7++
y4+
y5+
TVEEKVKK+XL_L
y6+
b2+
y6++
y7++
prec
y2+
y4++
b3+
y3+
b6++
y4+
y5+
b6+
